# Recombinant Tetrameric Neuraminidase Subunit Vaccine Provides Protection Against Swine Influenza A Virus Infection in Pigs

**DOI:** 10.3390/vaccines13080783

**Published:** 2025-07-23

**Authors:** Ao Zhang, Bin Tan, Jiahui Wang, Shuqin Zhang

**Affiliations:** Institute of Special Animal and Plant Sciences, Chinese Academy of Agricultural Sciences, Changchun 130112, China; zhangaoxuezz@163.com (A.Z.); tanbin@caas.cn (B.T.); x18545780919@163.com (J.W.)

**Keywords:** neuraminidase, subunit vaccine, Swine influenza A virus, antibodies

## Abstract

**Background/Objectives**: Swine influenza A virus (swIAV), a prevalent respiratory pathogen in porcine populations, poses substantial economic losses to global livestock industries and represents a potential threat to public health security. Neuraminidase (NA) has been proposed as an important component for universal influenza vaccine development. NA has potential advantages as a vaccine antigen in providing cross-protection, with specific antibodies that have a broad binding capacity for heterologous viruses. In this study, we evaluated the immunogenicity and protective efficacy of a tetrameric recombinant NA subunit vaccine in a swine model. **Methods**: We constructed and expressed structurally stable soluble tetrameric recombinant NA (rNA) and prepared subunit vaccines by mixing with ISA 201 VG adjuvant. The protective efficacy of rNA-ISA 201 VG was compared to that of a commercial whole inactivated virus vaccine. Pigs received a prime-boost immunization (14-day interval) followed by homologous viral challenge 14 days post-boost. **Results**: Both rNA-ISA 201 VG and commercial vaccine stimulated robust humoral responses. Notably, the commercial vaccine group exhibited high viral-binding antibody titers but very weak NA-specific antibodies, whereas rNA-ISA 201 VG immunization elicited high NA-specific antibody titers alongside substantial viral-binding antibodies. Post-challenge, both immunization with rNA-ISA 201 VG and the commercial vaccine were effective in inhibiting viral replication, reducing viral load in porcine respiratory tissues, and effectively mitigating virus-induced histopathological damage, as compared to the PBS negative control. **Conclusions**: These findings found that the anti-NA immune response generated by rNA-ISA 201 VG vaccination provided protection comparable to that of a commercial inactivated vaccine that primarily induces an anti-HA response. Given that the data are derived from one pig per group, there is a requisite to increase the sample size for more in-depth validation. This work establishes a novel strategy for developing next-generation SIV subunit vaccines leveraging NA as a key immunogen.

## 1. Introduction

Swine influenza A virus (swIAV), a member of the family Orthomyxoviridae and genus Alphainfluenzavirus, causes acute, febrile, and highly contagious respiratory disease in pigs [1]. Currently, the main SIVs prevalent worldwide include the H1N1, H1N2, and H3N2 subtypes [2]. The rapid evolution of SIV stems from the dual presence of avian- and human-type sialic acid receptors on porcine respiratory epithelial cells, enabling interspecies genetic reassortment of influenza viruses within swine [3]. This process generates novel reassortant strains with enhanced infectivity, pathogenicity, and transmissibility, posing substantial economic burdens on global livestock industries and significant public health risks [4,5,6]. Vaccination remains the optimal strategy to control SIV transmission in swine populations, limit viral recombination, and mitigate cross-species adaptation. However, frequent antigenic drift and shift in SIV necessitate the development of vaccines conferring durable, broad-spectrum protection against evolving strains.

Currently, influenza virus vaccine development primarily focuses on hemagglutinin (HA)-induced protective antibody responses. However, frequent antigenic changes in HA result in mismatches between vaccine strains and circulating variants, compromising vaccine efficacy [7,8]. The neuraminidase (NA), another major influenza surface glycoprotein, has emerged as a promising vaccine antigen for eliciting broad cross-protection [9,10,11]. NA naturally exists as a homotetramer formed by non-covalent association of four monomers. Its enzymatic activity facilitates viral release by cleaving sialic acid residues from host cell membranes via glycosidic bond hydrolysis, enabling virion dispersal and completing the infection cycle [12,13]. Critically, NA exhibits lower mutation rates than HA, and their antigenic evolution occurs independently [14,15]. This evolutionary stability allows NA-specific immunity to provide residual protection during HA antigenic drift [16]. NA induces the production of specific antibodies against NA, which play an important role in preventing influenza virus infection, inhibiting the spread of the virus, attenuating clinical symptoms, and enhancing the efficacy of vaccines [17,18,19]. Previous studies demonstrate that tetrameric NA conformation is essential for optimal immunogenicity. Tetrameric NA proteins elicit stronger immune responses and broader protection than monomeric forms. Intranasal co-administration of tetrameric rNA with c-di-GMP adjuvant protected mice against homologous and heterologous viral challenges, whereas trimeric HA provided limited heterologous protection [20,21]. Comparative analyses revealed that rNA-specific antibodies exhibit broader heterosubtypic binding capacity than HA-directed responses. These findings underscore NA’s unique advantages as a vaccine antigen for overcoming strain-specific limitations of HA-focused vaccines.

Current SIV vaccines predominantly rely on whole inactivated virus formulations produced in embryonated chicken eggs. However, serial egg-passaged SIV strains may acquire adaptive mutations at critical antigenic sites, compromising vaccine efficacy through antigenic mismatch [22]. Drawing parallels to human influenza vaccine development, cell culture-based SIV vaccine production is emerging as a viable alternative to conventional egg-dependent systems. The Chinese hamster ovary (CHO) cell expression system, widely utilized in biopharmaceutical production, enables stable expression of recombinant proteins with mammalian-like glycosylation patterns, yielding products with native structural and functional properties [23]. Comparative studies demonstrate that CHO-derived rH5HA induces higher neutralizing antibody titers and confers superior protective immunity against lethal challenge in murine models compared to Sf9 insect cell-expressed counterparts [24]. These findings highlight the potential of CHO platforms for subunit vaccine development. Subunit vaccines offer advantages including precise antigen targeting, enhanced safety profiles, and scalable manufacturing [25]. Notably, CHO-produced HA subunit vaccines (g127 + g138 HA) elicit cross-protective immunity against heterologous clades of highly pathogenic avian influenza H5N1 while generating robust neutralizing antibody responses [26]. This evidence underscores the transformative potential of CHO-based subunit vaccines in combating evolving influenza strains.

Previous influenza vaccine studies have predominantly utilized surrogate models such as mice [20,21,24]. However, swine serve as the natural host for SIV infection and pathogenesis, rendering them indispensable for evaluating SIV vaccine efficacy. Currently, only a handful of NA-based SIV vaccines have been licensed, such as Merck’s Sequivity IAV-S NA commercial vaccine. At present, limited data exist regarding the immunogenicity and protective efficacy of NA-based vaccines in porcine models. Furthermore, the immunogenic potential and protective capacity of tetrameric NA subunit vaccines remain underexplored in swine.

In this study, we assessed the immunogenicity and challenge protection of a tetrameric rNA subunit vaccine in swine. We systematically compared the immune profiles of rNA-ISA 201 VG and a commercial whole inactivated virus vaccine, alongside their protective efficacy against homologous viral challenge. Our findings found that tetrameric rNA subunit vaccination elicits robust immunogenicity and confers significant protection in swine, providing critical insights into NA-focused vaccine development for influenza control.

## 2. Materials and Methods

### 2.1. Cells, Virus, and Animals

Madin-Darby canine kidney (MDCK) cells (ATCC, CCL-34) were maintained in Minimum Essential Medium (MEM, Gibco, Waltham, MA, USA) containing 8% fetal bovine serum (FBS, Thermo Fisher Scientific, Waltham, MA, USA) at 37 °C with 5% CO_2_. ExpiCHO-S cells (Thermo Fisher Scientific, Waltham, MA, USA) were incubated at 36 °C, 8% CO_2,_ with a shake speed of 120 rpm on an orbital shaker platform (25 mm throw) using expression media (Thermo Fisher Scientific, Waltham, MA, USA). Swine influenza A virus (swIAV) strain A/swine/Jilin/25/2008(H1N1) was isolated and maintained by the Institute of Special Animal and Plant Sciences, Chinese Academy of Agricultural Sciences. The virus was propagated in MDCK cells, and the median tissue culture infectious dose (TCID_50_) of the virus was calculated by using the Reed and Muench method [27]. Nine 6-week-old pigs were purchased from a SIV-free pig farm.

### 2.2. Expression and Purification of rNA

**Figure 1:**
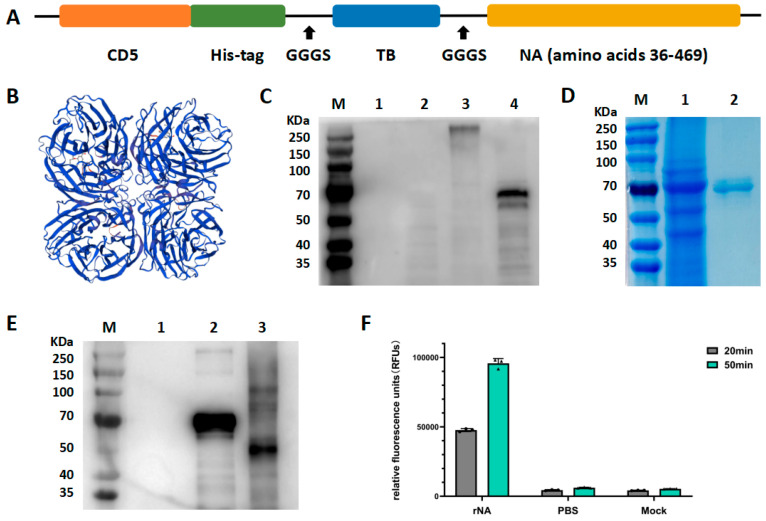
Construction, expression, and characterization of rNA. (**A**) Schematic structures of the soluble tetrameric rNA design. (**B**) Structure prediction of rNA protein using SWISS-MODEL. (**C**) Western blot analysis of rNA by blotting with the His-tag rabbit polyclonal antibodies. M, marker; lane 1, blank control (supernatant from untransfected ExpiCHO-S cells); lane 2, negative control (supernatant from ExpiCHO-S cells transfected with pcDNA3.1 vector); lane 3, supernatant from ExpiCHO-S cells transfected with pcDNA3.1-rNA recombinant construct (non-reducing conditions); lane 4, supernatant from ExpiCHO-S cells transfected with pcDNA3.1-rNA recombinant construct (reducing conditions). (**D**) Purified rNA was analyzed through SDS-PAGE performed under reducing conditions, followed by staining with Coomassie brilliant blue. M, marker; lane 1, pre-purified cells supernatant; lane 2, post-purified rNA protein. (**E**) N-glycosylation analysis of rNA protein in Western blot. M, marker; lane 1, blank control (supernatant from untransfected ExpiCHO-S cells); lane 2, rNA protein without PNGaseF treatment; lane 3, rNA protein with PNGaseF treatment. (**F**) Enzymatic activity analysis of rNA by neuraminidase assay.

The NA sequence was derived from A/swine/Jilin/25/2008(H1N1) (GenBank accession number: OQ740146). The cDNA of the NA ectodomain (amino acids 36–469) was cloned into the pcDNA3.1 expression plasmid with a CD5 secretion sequence, an N-terminal His-tag, and the tetrabranchion (TB) tetramerization domain (IINETADDIVYRLTVIIDDRYESLKNLITLRADR LEMIINDNVSTILA). A linker (GGGS) was fused in between the C-terminal of the TB tetramerization domain and the N-terminal of the NA ectodomain. The schematic structures of the soluble tetrameric design is illustrated in Figure 1A. The rNA was expressed by using the ExpiCHO Expression System (Thermo Fisher Scientific, Waltham, MA, USA) as described previously [28] and purified by using an Akta explorer chromatography system with a Ni Sepharose HisTrap excel column (GE Healthcare, Westborough, MA, USA). The purified protein samples were concentrated by using the centrifugal filter MWCO 30 kDa (Merck Millipore, Boston, MA, USA). Western blot and sodium dodecyl sulfate–polyacrylamide gel electrophoresis (SDS-PAGE) analyses were performed to monitor rNA expression, and to evaluate rNA purity during purification. Purified protein samples were denatured by mixing with reduced protein loading buffer and reduced protein loading buffer (EpiZyme, Shanghai, China), respectively, and heating at 95 °C for 10 min. Proteins were resolved in 10% SDA-PAGE and then stained with Coomassie brilliant blue. For Western blot analysis, the electrophoresis-resolved proteins in the SDS-PAGE gel were electro-transferred onto the PVDF membrane (Merck Millipore, Boston, MA, USA) and blocked in blocking buffer (EpiZyme, Shanghai, China) at room temperature for 30 min. The PVDF membrane was incubated with the His-tag rabbit polyclonal antibodies (EpiZyme, Shanghai, China) at a 1:3000 dilution overnight at 4 °C and washed with PBST 3 times. The membrane was then incubated with HRP-conjugated goat anti-rabbit IgG (EpiZyme, Shanghai, China) at a 1:8000 dilution for 2 h at room temperature, and the bands were revealed using ECL reagent (Beyotime, Shanghai, China). For glycosylation analysis, the rNA proteins were denatured and treated with PNGase F (New England BioLabs, Ipswich, MA, USA) and verified by 10% SDS-PAGE and Western blot.

### 2.3. Neuraminidase Activity Assay

The neuraminidase activity of purified rNA protein was tested using the neuraminidase assay kit (Sigma-Aldrich, St. Louis, MO, USA) according to the manufacturer’s protocol. Briefly, 20 μL of rNA protein was loaded into opaque black-bottom 96-well microplates, with parallel controls including PBS negative controls and substrate-free blank controls. Each well received 80 μL of premixed reaction solution. After homogenization on a horizontal shaker, the plates were incubated at 37 °C in the dark. Relative fluorescence intensity (RFU) was measured at excitation/emission wavelengths of 530/585 nm at 20 and 50 min post-reaction initiation using the microplate reader.

### 2.4. Preparation of Vaccine

The experimental vaccine was configured by mixing rNA antigen with the commercial adjuvant Montanide ISA 201 VG (Seppic, Paris, France) according to the manufacturer’s recommendations. The rNA protein was fully emulsified with the ISA 201 VG adjuvant at a volume ratio of water phase to oil phase of 45/55 at 31 °C. After cooling the adjuvanted vaccine to 20 °C, the water/oil/water (W/O/W) multiple emulsions were observed by microscope, and it was stored at 4 °C until use. The commercial vaccine used in the study was an inactivated vaccine for the H1N1 subtype of swine influenza virus (Keqian Biology, Wuhan, China).

### 2.5. Immunization and Viral Challenge Experiments

**Figure 2:**
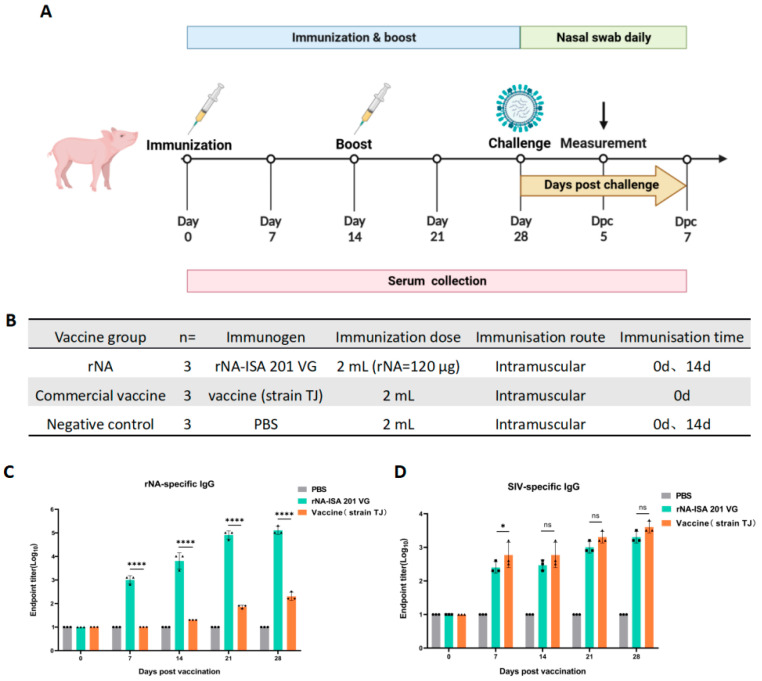
Humoral immune responses to rNA-ISA 201 VG vaccination in pigs. (**A**) Vaccination and challenge regimen. Pigs in the three groups received booster immunization on day 14 after primary immunization using the same dose and route (the commercial vaccine group followed the manufacturer’s protocol without booster immunization). Sera were collected for antibody testing on days 0, 7, 14, 21, and 28 after primary immunization. Viral challenge was performed 14 days post-booster. Throughout the challenge period, clinical symptoms, rectal temperature, and body weight were monitored daily for 7 consecutive days, with nasal swabs concurrently collected for viral isolation. On day 5 post-challenge, one pig from each group was euthanized for necropsy. Trachea, lung, and hilar lymph node tissues were collected for viral load quantification and histopathological analysis. (**B**) Pigs were randomly divided into three groups, including the rNA group, the commercial vaccine group, and the negative control group. Each group received intramuscular injections of equivalent volumes of rNA-ISA 201 VG complex, commercial vaccine (strain TJ), or PBS, respectively. (**C**,**D**) Titration of (**C**) rNA-specific serum IgG and (**D**) SIV-specific serum IgG in ELISA. The reciprocals of the highest dilution of serum with an optical density greater than 2.1 times the background values were defined as the endpoint titers. Log10-transformed groups were compared using two-way ANOVA with a Tukey posttest. Significance levels were annotated as follows: ns (*p* value > 0.05), * (*p* value < 0.05), and **** (*p* value < 0.0001).

The immunization and challenge experiment in pigs was conducted as outlined in Figure 2A, with experimental groups detailed in Figure 2B. Pigs were randomly allocated into three groups (*n* = 3): the rNA group, commercial vaccine group, and negative control group. Prior to immunization, all pigs were confirmed to be free of upper respiratory symptoms and tested negative for SIV antigens/antibodies and common respiratory pathogens. Immunization was administered via intramuscular injection in the neck region (120 µg per dose). A booster immunization was performed on day 14 post-primary immunization using the same dosage and route (the commercial vaccine group followed the manufacturer’s protocol without booster). Post-immunization adverse reactions were monitored daily for 7 days. Blood samples were collected at 0, 7, 14, 21, and 28 days post-primary immunization, centrifuged at 1000× *g* for 15 min, and sera were stored at −20 °C for subsequent analyses.

Fourteen days post-booster immunization, challenge experiments were initiated. Pigs were intranasally challenged with the strain A/swine/Jilin/25/2008(H1N1) (10^4.5^ TCID_50_). Following viral challenge, clinical symptoms were monitored and recorded daily for 7 days across all groups. Body weight and rectal temperature measurements were conducted concurrently during this observation period. Nasal swabs were collected daily for 7 days to evaluate viral shedding. On day 5 post-challenge, one pig per group was euthanized for necropsy. Trachea, lung, and hilar lymph node tissues were collected for histopathological assay and viral replication analysis.

### 2.6. Antibody Detection

rNA- and SIV-specific IgG antibody titers in sera from the immunized animals were determined by indirect ELISA. The 96-well ELISA plates were coated with 100 µL rNA protein (2 µg/mL) or SIV (2.5 µg/mL) overnight at 4 °C. For rNA-specific IgG antibody detection, the plates were blocked with 200 µL 5% skim milk at 37 °C for 2 h. For SIV-specific IgG antibody detection, the plates were blocked with 200 µL 10% skim milk overnight at 4 °C. Serum samples were serially diluted and added to the plates, followed by incubation at 37 °C for 1 h. After three washes with PBST (1×PBS with 0.05% Tween 20), the plates were incubated with 100 µL HRP-conjugated rabbit anti-pig IgG (Bioss, Beijing, China) at a 1:5000 dilution for 30 min at 37 °C and developed with TMB substrate (Solarbio, Beijing, China) for 15 min at 37 °C. Absorbance was measured at 450 nm using the microplate reader. The reciprocals of the highest dilution of serum with an optical density greater than 2.1 times the background values were defined as the endpoint titers.

### 2.7. Viral Isolation and Hemagglutination (HA) Assay

Viral isolation from nasal swab samples was performed to assess viral shedding differences between vaccinated and unvaccinated animals. Nasal swabs were vortexed in 2 mL MEM containing penicillin–streptomycin, centrifuged at 1000× *g* for 10 min at 4 °C, and filtered through 0.22 μm membranes. The supernatants were aliquoted and stored at −80 °C for viral isolation. Viral isolation was conducted in MDCK cells by adding 200 µL of nasal swab supernatant to MDCK cell cultures on 24-well plates supplemented with 300 µL of serum-free MEM containing 2 μg/mL of TPCK trypsin, and incubation for 72 h at 37 °C with 5% CO_2_. Post-incubation, the wells were examined microscopically for cytopathic effect (CPE), and the HA titer of the isolated virus was determined by HA assays with 1% chicken erythrocytes.

Twofold serial dilutions of viral isolates (50 μL/well) were prepared in V-bottom 96-well microplates. Equal volumes of 1% erythrocyte suspension were added, with negative controls (PBS + erythrocytes without viral inoculum) included in the final column. After the addition of samples, the plate was gently agitated and incubated at room temperature for 30 min, and the results were determined when control wells showed distinct button-shaped sedimentation. The HA titer was defined as the highest dilution causing complete erythrocyte agglutination, and the HA titer ≥ 4 log2 was judged as positive.

### 2.8. Determination of Virus Load

On day 5 post-challenge, one pig per group was euthanized for necropsy. Trachea, lung, and hilar lymph node tissues were collected for viral load quantification. Total RNA was extracted from tissues using TRIzol reagent (Thermo Fisher Scientific, Waltham, MA, USA), followed by reverse transcription to cDNA with All-in-One RT MasterMix (Abm, New York, NY, USA) according to the manufacturer’s instructions. Viral load in infected tissues was quantified using real-time quantitative PCR (qPCR). Viral genes were amplified using 2× RealStar Fast SYBR qPCR Mixture (GenStar, Beijing, China), with β-actin employed as the reference gene to normalize inter-sample variations. Two sets of primers designed in-house were employed for qPCR amplification, with sequences as follows: SIV-F, TGGATGGACGGAA AC; SIV-R, ACGCCACAGAAAGAAA; β-actin-F,CATTGCTGACAGGATGCAGAAGG; β-actin-R,TGCTGGAAGGTGGACAGTGA GG.

### 2.9. Histopathology Assay

For histopathology assay, trachea, lung, and hilar lymph node tissues were fixed in 10% neutral buffered formalin for 48 h, dehydrated, paraffin-embedded, and sectioned at 4 μm thickness. Tissue sections were deparaffinized in xylene, rehydrated through graded ethanol series, and stained with hematoxylin and eosin (H&E) for microscopic examination.

### 2.10. Statistical Analyses

The statistical analyses were performed using GraphPad Prism 8 software (San Diego, CA, USA). For all analyses, *p* values were analyzed with a two-tailed *t*-test or two-way ANOVA, with statistical significance defined as *p* value < 0.05. Significance levels were annotated as follows: ns (*p* value > 0.05), * (*p* value < 0.05), ** (*p* value < 0.01), *** (*p* value < 0.001), and **** (*p* value < 0.0001).

## 3. Results

### 3.1. Generation and Characterization of rNA

The tetrameric assembly state of rNA is critical for the catalytic activity of NA and the antigenicity of the NA head domain. The tetrameric conformation of rNA was stabilized by fusing the ectodomain of A/swine/Jilin/25/2008 (H1N1) with a TB tetramerization domain from the bacterium Staphylothermus marinus (Figure 1A). A CD5 signal peptide was added to the N-terminal of the NA ectodomain to enable secretory expression of rNA, and a His tag was added to facilitate purification. The tertiary structure of the designed and constructed rNA was predicted using SWISS-MODEL (Figure 1B). The recombinant construct was transiently transfected into ExpiCHO-S cells for protein production. Western blot performed under reducing conditions showed that rNA migrated as a single band with a molecular mass of approximately 60–70 KDa, and no specific bands were seen in either negative or blank controls (Figure 1C). Non-reducing conditions produced a single band at a molecular mass of about 250 KDa, approximately fourfold the molecular weight under reducing conditions, indicating that the rNA protein was successfully secreted and assembled into a tetramer in ExpiCHO-S cells (Figure 1C). Ni affinity chromatography was used to purify the recombinant proteins from culture supernatants, and SDS-PAGE performed under reducing conditions showed a purified protein band at a molecular mass of about 60–70 kDa with minimal heteroproteins (Figure 1D).

To validate N-glycosylation, PNGase F-treated rNA exhibited a reduced molecular weight shift from 60–70 kDa to 55 kDa by Western blot under reducing conditions, confirming extensive N-linked glycosylation of the tetrameric rNA expressed in ExpiCHO-S cells (Figure 1E). Typically, enzymatic activity is used as a measure of the correct conformation of an NA antigen. In our study, the neuraminidase activity of rNA was observed experimentally, indicating that rNA was correctly folded (Figure 1F).

### 3.2. Immunogenicity of rNA in Swine

To evaluate the immunogenicity of tetrameric rNA protein in pigs, pigs were randomly divided into three groups (*n* = 3): immunized with equal doses of rNA-ISA 201 VG, commercial vaccine (strain TJ), or PBS, respectively (Figure 2B). Serum samples were collected at multiple timepoints post-immunization to monitor the duration of the antibody response (Figure 2A). rNA-specific IgG levels were quantified via indirect ELISA to assess the dynamics of rNA-binding antibodies (Figure 2C). All pigs were seronegative for NA-specific IgG before vaccination on day 0 (Figure 2C). The rNA group induced the production of rNA-specific conjugated IgG antibodies in pigs as soon as the first immunization, and antibody titres increased and reached high levels over time, whereas NA-specific IgG was detected in the commercial vaccine group until 14 d after the initial immunization (Figure 2C). The rNA-specific antibody titres of the rNA group were 10^3.8^ at 14 d after the first immunization, and the antibody titres could reach 10^5.1^ at 28 d after the first immunization, which was statistically significant (*p* < 0.0001) compared with the commercial vaccine group (Figure 2C). These results found that rNA-ISA 201 VG elicited robust and sustained humoral immunity in pigs. For SIV-specific IgG, the ELISA results showed that both the rNA group and the commercial vaccine group induced the production of specific binding IgG antibodies against SIV in pigs after the first immunization, and the titres of SIV-specific antibodies increased over time, and there was no statistically significant difference in SIV-specific antibodies induced by the two groups except for the 7 d after the first immunization (*p* > 0.05) (Figure 2D).

### 3.3. Immunization with rNA-ISA 201 VG Reduced Clinical Signs in Pigs Caused by SIV Infection

**Figure 3:**
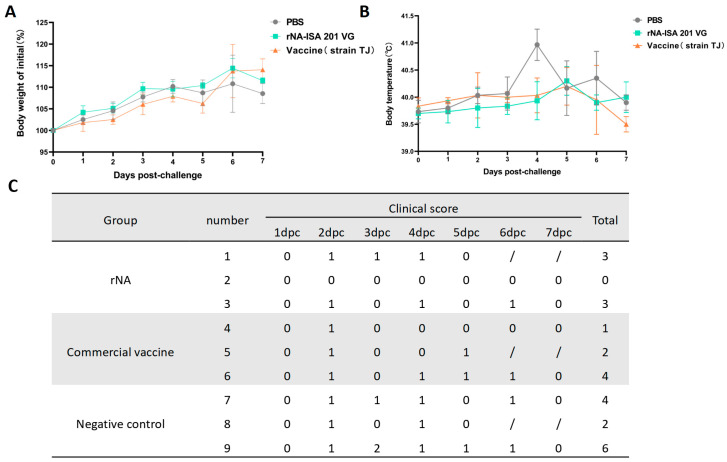
Immunization with rNA-ISA 201 VG and commercial vaccine (strain TJ) reduced clinical signs in pigs caused by SIV infection. (**A**) Changes in body weight in pigs challenged with SIV for 7 days after virus infection. (**B**) Changes in rectal temperature in pigs challenged with SIV for 7 days after virus infection. (**C**) The clinical symptoms score of pigs challenged with SIV for 7 days after virus infection. Coughing/nasal discharge/sneezing/dyspnea are each scored 1 mark. “0” indicates no clinical symptoms. “/” indicates no content. Pigs No. 1, No. 5, and No. 8 were examined by autopsy on day 5 after the challenge, so there were no records of clinical symptoms on day 6 and day 7 after the challenge.

Clinical signs, rectal temperature, and body weight of the pigs in each group were monitored daily for 7 consecutive days post-challenge. No significant weight loss was observed across groups; however, the rNA group exhibited the highest percentage of weight gain compared with the average weight before the challenge (Figure 3A). For the post-challenge changes in body temperature, pigs in the negative control group developed elevated body temperatures starting at day 2 post-challenge, peaking at 40.9 °C on day 4, followed by a gradual decline to pre-challenge average body temperature on day 7 (Figure 3B). In contrast, both rNA and commercial vaccine groups showed milder febrile responses, with peak temperatures of 40.3 °C and 40.2 °C, respectively, observed on day 5 (Figure 3B). The results of clinical symptom scoring revealed that SIV-associated clinical signs in all groups of pigs from day 2 post-challenge, with the exception of pig No. 2 in the rNA group. Pigs immunized with rNA-ISA 201 VG and commercial vaccine were relatively milder in terms of the duration and severity of clinical symptoms compared to the negative control (Figure 3C).

### 3.4. Immunization with rNA-ISA 201 VG Reduced Viral Replication in Infected Pigs

**Figure 4:**
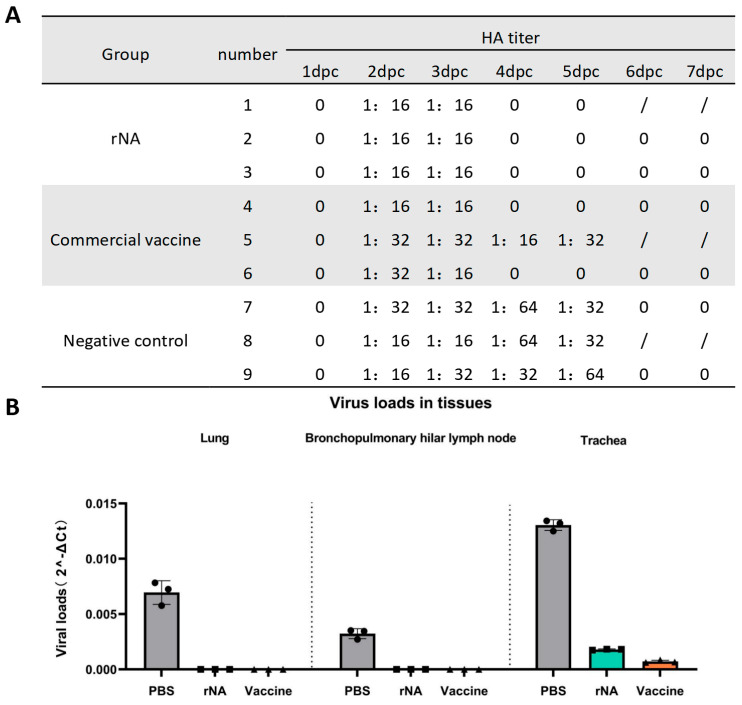
Immunization with rNA-ISA 201 VG and commercial vaccine (strain TJ) reduced viral replication in infected pigs. (**A**) Results of viral isolation and HA titers of nasal swabs from pigs challenged with SIV. HA titers ≥ 1:16 are determined as positive for virus isolation. “0” indicates negative for viral isolation. Pigs No. 1, No. 5, and No. 8 were dissected on day 5 after the challenge, so there were no results of virus isolation from nasal swabs on day 6 and day 7 after the challenge. (**B**) Viral loads in the respiratory tissues of pigs with SIV challenged on day 5 post-challenge. The data represent the results of repeat measurements for one pig per group.

Nasal swab samples were collected daily for 7 days post-challenge for viral isolation in MDCK cells and HA titer determination using 1% chicken erythrocytes. All groups of pig nasal swabs were positive for viral isolation and HA titers ≥ 1:16 on day 2 post-challenge with the homologous virus (Figure 4A). Viral replication in nasal swabs was detectable from day 2 post-challenge in all groups (Figure 4A). Notably, neither the rNA group nor the commercial vaccine group (except for pig No. 5) yielded isolatable virus in nasal swabs after day 4 post-challenge, whereas the negative control group maintained viral replication until day 6 post-challenge (Figure 4A). The above results indicated that immunization with rNA-ISA 201 VG and commercial vaccine could effectively inhibit SIV replication and shorten the viral shedding time of the pigs after the challenge, and the effect of rNA-ISA 201 VG was better than that of the commercial vaccine.

One pig in each group was selected for autopsy on day 5 post-challenge. Trachea, lung, and hilar lymph node tissues were collected for viral load quantification. On day 5 post-challenge, no SIV was detected in lung or hilar lymph node tissues from the rNA and commercial vaccine groups, whereas SIV could be detected in the lungs and hilar lymph nodes of the PBS control group (Figure 4B). The rNA and commercial vaccine groups had significantly lower viral loads in the lungs and hilar lymph compared with the PBS control group (*p* < 0.001) (Figure 4B). In addition, the viral load detected in the trachea was also significantly lower in the rNA and commercial vaccine groups than in the PBS control group (*p* < 0.0001), while the viral load in the commercial vaccine group was significantly lower than that in the rNA group (*p* < 0.001) (Figure 4B). These findings found that while both vaccines effectively reduced viral loads in respiratory tissues, the commercial vaccine showed superior efficacy in tracheal viral clearance.

### 3.5. Immunization with rNA-ISA 201 VG Reduced Tissue Damage and Pathological Changes in Pigs Caused by Viral Infection

**Figure 5:**
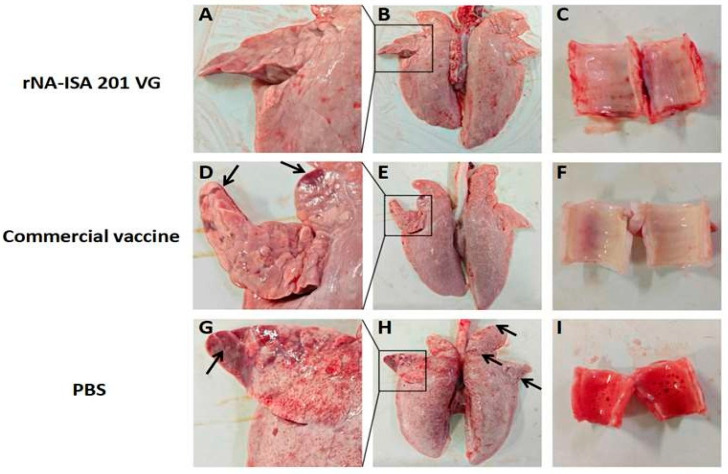
Macroscopic Lesions of respiratory tissues of pigs with SIV challenge. (**A**–**C**) The lung and trachea of pigs in rNA group. (**D**–**F**) The lung and trachea of pigs in the commercial vaccine group. (**G**–**I**) The lung and trachea of pigs in the PBS negative group. The images depict the macroscopic lesion results for each group, based on one pig per group. Black arrows indicate substantial lesions in the lungs.

One pig from each group was selected for autopsy on day 5 post-challenge, and the lesions in the trachea and lungs of the infected pigs were observed. Ocular observation of the lesions revealed diffuse hemorrhage in the lung tissues of the pigs in the PBS negative control group, with extensive substantial lesions in the right acinar lobe, and patchy substantial lesions in the left cardiac lobe, the left acinar lobe, and the right septal lobe, with some of the solid lesions masked by diffuse congestion (Figure 5A,B). Large amounts of red foam were seen in the pig trachea (Figure 5C). In contrast, pigs in the rNA group had no red foam in the trachea, only a few hemorrhages were seen, and the lung tissue was basically normal, with only slight punctate solid lesions on the ventral surface of the right heart lobe (Figure 5G–I). In the commercial vaccine group, the pigs in the right apical lobe, the right heart lobe, and the ventral surface of the left apical lobe showed punctate solid lesions, and the trachea showed no obvious lesions (Figure 5D–F).

**Figure 6:**
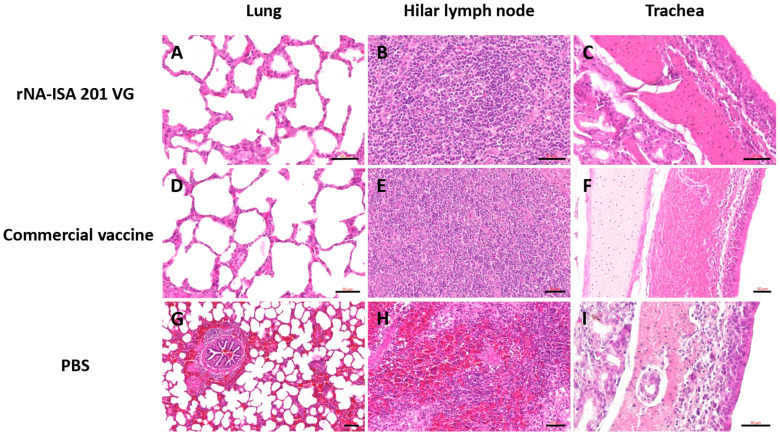
The histopathology assay of respiratory tissues of pigs with SIV challenge. (**A**–**C**) The HE of lung, hilar lymph node, and trachea in the rNA group. Scale bar = 50 μm. (**D**–**F**) The HE of lung, hilar lymph node, and trachea in the commercial vaccine group. Scale bar = 50 μm. (**G**) Significant hemorrhage and inflammatory cell infiltrate in the lungs of the PBS negative group. Scale bar = 100 μm. (**H**) Significant hemorrhage in the bronchopulmonary hilar lymph node of the PBS negative group. Scale bar = 50 μm. (**I**) Tracheal cilia shedding, with infiltration of macrophages and lymphocytes in the trachea of the PBS negative group. Scale bar = 50 μm. The images depict the results based on one pig per group.

The trachea, lung, and hilar lymph node tissues were subjected to HE staining for pathological evaluation. The results of pathological analysis showed that the lungs of pigs in the PBS negative control group had obvious lesions, with unclear alveolar structures, accompanied by a large number of inflammatory cell infiltrations, obvious hemorrhage and bruising, and the alveolar septum and alveolar lumen were filled with a large number of red blood cells (Figure 6G). It was also seen that some of the fine bronchial epithelial cells of the pig were detached, with lymphocytes oozing out around the tubes, forming a tubular sleeve (Figure 6G). The number of columnar epithelial cells in the mucosa of some regions of the trachea was reduced, and in some cases, cell necrosis, nuclear condensation, and cilia detachment were seen, and in some cases, macrophages and lymphocytes were seen to be exuding from the submucosa (Figure 6I). Hemorrhage was evident in most areas of porcine hilar lymph nodes, and a large number of erythrocytes were seen oozing from the cortex and medulla (Figure 6H). In contrast, the histological structure of the lungs of pigs in the rNA group and the commercial vaccine group was basically normal, with clear alveolar structure, regular arrangement, and only a small amount of inflammatory cell infiltration (Figure 6A,D). Lung hilar lymph nodes were clear in structure, with dense lymphocytes (Figure 6B,E). The columnar epithelial cells of the tracheal mucosa had a clear structure, with a small number of macrophages and lymphocytes exuding from the submucosa (Figure 6C,F). These results indicate that both porcine immunization with rNA-ISA 201 VG and commercial vaccines can effectively reduce tissue damage caused by homologous virus infection.

## 4. Discussion

Current vaccines against SIV are mostly whole-virus inactivated and depend on chicken embryos for production. Subunit vaccine platforms employing diverse protein expression systems have emerged as promising alternatives to the traditional chicken embryo-based vaccine production, demonstrating substantial potential in combating major infectious diseases. In recent years, several studies have revealed the potential advantages of influenza virus NA proteins as a vaccine antigen. Recent studies showed that intranasal immunization with tetrameric rNA protein paired with c-di-GMP adjuvant conferred protection against both homologous and heterologous viral challenges in mice [12]. Furthermore, other studies have shown that tetrameric NA proteins provide protection against homologous influenza virus attack as well as subtype-specific cross-protection in mice [21]. Most previous influenza vaccine studies have been conducted in alternative animals such as mice, and pigs, as target animals for SIV infection and pathogenesis, are irreplaceable animal models for studying the efficacy of SIV vaccines.

In this study, we evaluated the immunogenicity and protective efficacy against challenge of a tetrameric rNA subunit vaccine in a swine model. We constructed and expressed structurally stable soluble tetrameric rNA in which NA was derived from A/swine/Jilin/25/2008 (H1N1) and mixed it with ISA 201 VG adjuvant to prepare the subunit vaccine. To assess the immunogenic potential of rNA, pigs were immunized twice with rNA-ISA 201 VG at 14-day intervals. The commercial vaccine group and the PBS negative control group were immunized with equal doses of whole inactivated virus vaccine and PBS, respectively. ELISA analysis revealed that both rNA-ISA 201 VG and the commercial vaccine stimulated robust humoral responses in swine. Immunization with rNA-ISA 201 VG was able to stimulate a specific immune response in pigs, inducing the production of high levels of NA-specific IgG antibodies, whereas pigs immunized with the commercial vaccine had very weak anti-NA antibody titres. In addition, we compared the ability of the rNA group to stimulate the production of virus-specific binding antibodies in pigs with that of the commercial vaccine group. The results showed that the rNA group could produce specific antibodies against SIV, and the commercial vaccine induced higher SIV-specific IgG antibody levels than the rNA vaccine. However, due to the large individual variability between the groups, the difference in virus-specific binding antibodies between the two groups was not statistically significant, and further studies are needed in the future.

Next, we assessed the protective efficacy of rNA-ISA 201 VG against homologous viral challenge in the swine model. Pigs were intranasally challenged with A/swine/Jilin/25/2008(H1N1) (10^4.5^ TCID_50_) 14 days post-booster immunization. The protective effect of rNA-ISA 201 VG was evaluated by observation of clinical signs, viral isolation from nasal swabs, viral load quantification in tissues, and histopathological analysis of the pigs after challenge. In comparison to a randomly selected pig from the PBS negative control group, a randomly selected pig from the rNA group exhibited reduced viral load in trachea, lungs, and hilar lymph node tissues, attenuated clinical symptoms, suppressed SIV replication, and shortened viral shedding duration. Given that the data are derived from one pig per group, there is a requisite to increase the sample size for more in-depth validation. Previous studies in mouse models demonstrated complete protection against homologous influenza challenge with tetrameric rNA immunization [12]. However, in this swine model, neither rNA-ISA 201 VG nor the commercial vaccine provided complete protection against challenge, as evidenced by residual viral isolation from nasal swabs post-challenge. These findings underscore the irreplaceable role of swine in evaluating SIV vaccine efficacy. In addition, the protective efficacy of the tetrameric rNA protein subunit vaccine still needs to be studied more thoroughly in terms of different administration routes, antigen doses, and adjuvant formulations, so as to find out the immunization conditions that can provide the best protective effect.

Body weight index is an important indicator for evaluating the tolerance of immunized animals against viral challenge. Most of the previous influenza vaccine studies were conducted on replacement animals such as mice, which were seen to lose significant weight or even die [29,30]. In immunization and virulence studies on pigs, body weight indicators are less frequently used. In the present study, immunization and challenge were conducted on SIV-infected and diseased target animals, and it was found that pigs did not show significant weight loss after SIV challenge, probably due to the differences in virulence factors of SIV strains in pigs and replacement animals. In this study, SIV challenge marginally impaired weight gain in the immunized versus PBS control group, though systematic comparisons between challenged and unchallenged pigs were not conducted. Therefore, subsequent expansion of the sample size is needed for more in-depth validation.

In addition, histopathological analysis further revealed extensive pulmonary lesions in a randomly selected pig from the PBS control group, characterized by diffuse hemorrhage, large consolidated areas, and dense inflammatory cell infiltration. Hilar lymph nodes exhibited marked hemorrhage and erythrocyte extravasation in cortical/medullary regions. Tracheal mucosa displayed reduced columnar epithelial density with cellular necrosis, pyknosis, and ciliary loss. In contrast, a randomly selected pig from both the rNA-ISA 201 VG group and the commercial vaccine group maintained near-normal tracheopulmonary architecture. Minor pathological changes included sporadic macrophage and lymphocyte infiltration in tracheal submucosa and punctate consolidation foci with scant inflammatory cells in pulmonary tissues. Hilar lymph nodes showed no significant pathology. These findings found that rNA-ISA 201 VG immunization effectively attenuates homologous virus-induced tissue damage to levels comparable with commercial vaccine efficacy. From the histopathological observation of the tracheal tissue, it was shown that the commercial vaccine presented better immune protection compared to the rNA-ISA 201 VG group, so more studies including the impact of antigen formulation, immune kinetics, or adjuvant effects still needed.

## 5. Conclusions

The rNA-ISA 201 VG immunization conferred substantial protection against homologous SIV challenge in swine. Immunization with rNA-ISA 201 VG effectively suppressed viral replication, shortened viral shedding duration, and significantly reduced viral loads in trachea, lungs, and hilar lymph node tissues of pigs, and attenuated tissue damage lesions caused by homologous viral infections. Although a robust homologous protective effect has been exhibited, the limited sample size necessitates an expansion of the sample size for more in-depth validation. Furthermore, its cross-protective capacity against heterologous viruses requires further investigation. These findings contribute to a better understanding of the role of anti-NA immunity in the prevention of SIV infection and disease.

## 6. Limitations and Future Work

The limitations of this study include the relatively small number of pigs utilized in the experiments. This investigation serves as a preliminary examination of the protective efficacy of the vaccine. In future studies, we intend to increase the sample size for more comprehensive investigations, including studies on the vaccine’s antigen formulation, immune kinetics, adjuvant effects, and its protective efficacy against heterologous viruses.

## Data Availability

The original contributions presented in this study are included in the article. Further inquiries can be directed to the corresponding author.
